# A Novel Schizophrenia Diagnostic Model Based on Statistically Significant Changes in Gene Methylation in Specific Brain Regions

**DOI:** 10.1155/2020/8047146

**Published:** 2020-02-12

**Authors:** Donghua Zou, Yufen Qiu, Rongjie Li, Youshi Meng, Yuan Wu

**Affiliations:** ^1^Department of Neurology, The Fifth Affiliated Hospital of Guangxi Medical University, Nanning, Guangxi 530022, China; ^2^Maternal and Child Health Hospital and Obstetrics and Gynecology Hospital of Guangxi Zhuang Autonomous Region, Nanning, Guangxi 530003, China; ^3^Department of Neurology, The First Affiliated Hospital of Guangxi Medical University, Nanning, Guangxi 530021, China

## Abstract

**Objective:**

The present study identified methylation patterns of schizophrenia- (SCZ-) related genes in different brain regions and used them to construct a novel DNA methylation-based SCZ diagnostic model.

**Methods:**

Four DNA methylation datasets representing different brain regions were downloaded from the Gene Expression Omnibus. The common differentially methylated genes (CDMGs) in all datasets were identified to perform functional enrichment analysis. The differential methylation sites of 10 CDMGs involved in the largest numbers of neurological or psychiatric-related biological processes were used to construct a DNA methylation-based diagnostic model for SCZ in the respective datasets.

**Results:**

A total of 849 CDMGs were identified in the four datasets, but the methylation sites as well as degree of methylation differed across the brain regions. Functional enrichment analysis showed CDMGs were significantly involved in biological processes associated with neuronal axon development, intercellular adhesion, and cell morphology changes and, specifically, in PI3K-Akt, AMPK, and MAPK signaling pathways. Four DNA methylation-based classifiers for diagnosing SCZ were constructed in the four datasets, respectively. The sample recognition efficiency of the classifiers showed an area under the receiver operating characteristic curve of 1.00 in three datasets and >0.9 in one dataset.

**Conclusion:**

DNA methylation patterns in SCZ vary across different brain regions, which may be a useful epigenetic characteristic for diagnosing SCZ. Our novel model based on SCZ-gene methylation shows promising diagnostic power.

## 1. Introduction

Schizophrenia (SCZ) is a serious mental illness [1]. The World Health Organization estimates the global lifetime prevalence of SCZ at 3.8–8.4% [2]. SCZ is a severe psychosis induced by multiple factors and it manifests as a clinical syndrome with many symptoms. The course of the disease can include repeated relapses that aggravate disease and reduce quality of life. Some patients suffer from depression or mental disability [3]. Currently, clinical diagnosis of SCZ is based on the diagnostic scale of International Mental Disorders Classification [4]. The heterogeneous nature of SCZ pathogenesis has made it impossible so far to identify a single, reliable diagnostic biomarker or model.

Previous studies have shown that epigenetic changes may be related to the pathology of SCZ [5]. DNA methylation is the most stable epigenetic modification, and it can lead to changes in phenotype although the DNA sequence remains unchanged [6, 7]. DNA methylation may affect neuronal activity, transcriptional output, and synaptic function. Thus, methylation may be important in the pathology of SCZ [8]. Abnormal methylation of some genes, such as *DRD*2 [9], *DLGAP*2 [10], or *COMT* [11], may be associated with the occurrence and development of SCZ. In addition, a few DNA methylation-based classifiers for SCZ diagnosis have been reported [12, 13]. However, these studies were limited because they relied more on statistical associations without in-depth analysis of biological function.

In the present study, we identified common differentially methylated genes (CDMGs) shared across different brain regions in SCZ patients. We used the 10 CDMGs involved in the greatest number of neurological or psychiatric-related biological processes to construct a DNA methylation-based classifier for SCZ diagnosis. This method may be more biologically relevant than previous ones and may provide new insights to guide future research in SCZ.

## 2. Materials and Methods

### 2.1. DNA Methylation in SCZ

**Figure 1:**
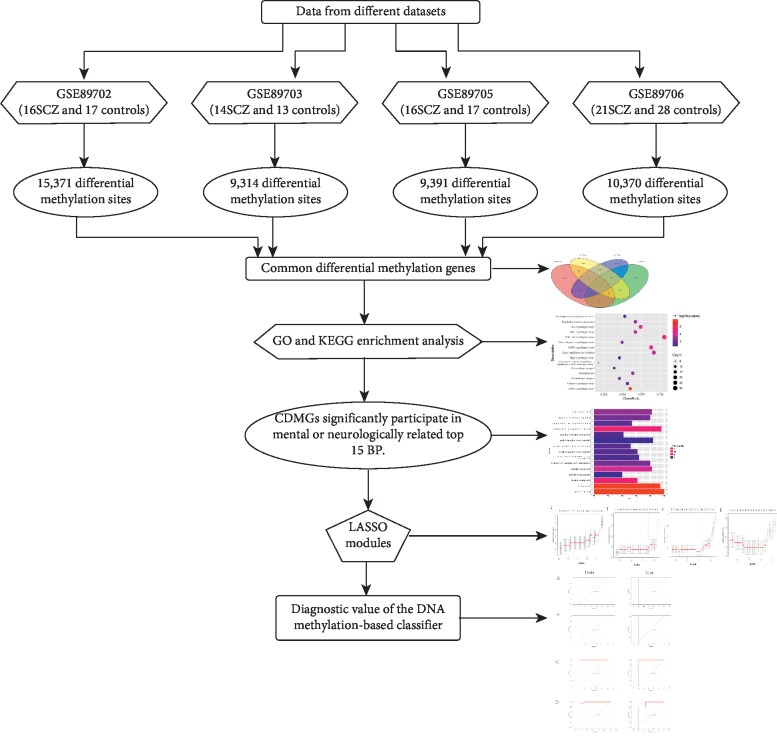
Flowchart of the present study. Abbreviations: SCZ, schizophrenia; CDMGs, common differential methylated genes; GO, Gene Ontology; KEGG, Kyoto Encyclopedia of Genes and Genomes; LASSO, least absolute shrinkage and selection operator.

In the present study, four SCZ methylation datasets (GSE89702, GSE89703, GSE89705, and GSE89706) were downloaded from the Gene Expression Omnibus database (https://www.ncbi.nlm.nih.gov/geo/). DNA was isolated from postmortem brain samples. GSE89702 was derived from cerebellum from the Douglas Bell-Canada Brain Bank, and it included 16 SCZ samples and 17 normal controls. GSE89703 was derived from hippocampus, and it included 14 SCZ samples and 13 normal controls. GSE89705 was derived from striatum, and it included 16 SCZ samples and 17 normal controls. GSE89706 was derived from striatum from the London Brain Bank for Neurodegenerative Disorders, and it included 21 SCZ samples and 28 normal controls. The platform was GPL13534 and included each probe, the position on the chromosome, and the corresponding gene name of each probe. The normalized methylation data matrix was shown as beta values (ranging 0 to 1) of each probe with probe ID in row and patient ID in column. The workflow of the present study is shown in Figure 1.

### 2.2. Differential Methylation Analysis

Although GSE89705 and GSE89706 were both taken from the region of striatum, they were not combined because we did not know if there is any difference in the processing of samples between the two brain banks and that it is possible that combining datasets may result in some residual inflation according to a published study [14]. Thus, differential methylation analysis was performed separately in the four datasets using the limma package [15] in R software. The size of samples in each dataset was relatively small, and thus, the significance of differential methylation sites may be relatively low. If rigorously *P* value filtering, genes with potential biological function may be filtered out. Therefore, in the present study, differences with a *P* value <0.05 were considered significant.

### 2.3. Enrichment Analysis

To explore the biological functions of CDMGs that may be related to SCZ, Kyoto Encyclopedia of Genes and Genomes (KEGG) and Gene Ontology (GO) enrichment analyses were performed using the ClusterProfiler package [16] in R. GO terms and KEGG pathways with *P* values <0.05 were considered significant.

### 2.4. Variable Selection and LASSO Classifier

Differentially methylated genes were involved in larger numbers of neurological or psychiatric-related biological processes and their methylation levels are thought to be more likely associated with SCZ than genes involved in fewer such processes. The differential methylation genes are different in different brain regions, and the differential methylation sites of the same gene in different brain regions may also be different. So we tried to build a brain-specific methylation-based classifier for SCZ. The corresponding different methylation sites of ten CDMGs involved in the most neurological or psychiatric-related biological processes were used to construct a diagnostic model of SCZ. These samples of the four datasets were randomly assigned to the training set (75%) and test set (25%), respectively. The four training sets were, respectively, used to select variables (different methylation sites) for establishing a DNA methylation-based classifier, and the test sets were used to validate the four classifiers. The glmnet package [17] in R used the least absolute shrinkage and selection operator (LASSO) [18] was used to select variables and construct a DNA methylation-based diagnostic classifier.

### 2.5. Evaluation of Methylation-Based SCZ Diagnostic Model

The diagnostic performance of the DNA methylation-based SCZ diagnostic model was evaluated by accuracy, sensitivity, specificity, positive predictive value, negative predictive value, and area under the receiver operating characteristic curve (AUC) as analyzed in the pROC package [19] in R software.

## 3. Results

### 3.1. SCZ-Related CDMGs in Different Brain Regions

**Figure 2:**
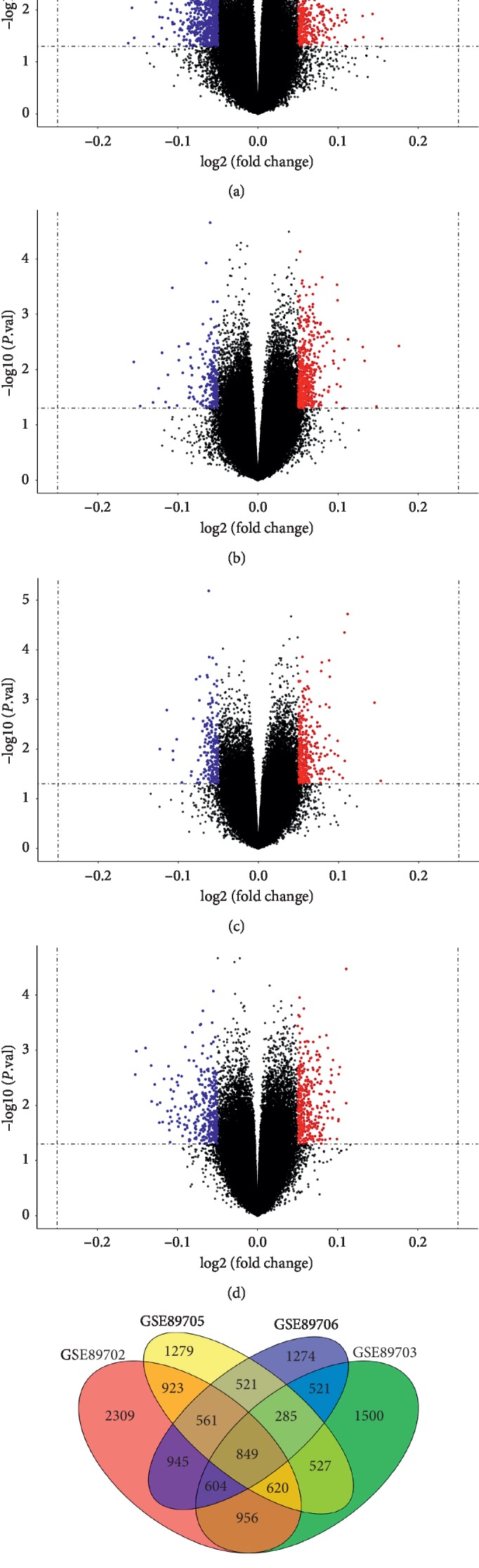
Volcano plots of differentially methylated genes from each dataset. (a) GSE89702. (b) GSE89703. (c) GSE89705. (d) GSE89706. Red represents hypermethylation in SCZ relative to controls; blue, hypomethylation; and grey, no significant difference in methylation levels. (e) Venn diagram of differentially methylated genes amongst the four datasets.

We assessed DNA methylation characteristics from the four datasets taken from different regions of the brain. Compared with control groups from the respective datasets, we identified 7887 hypermethylated positions and 7484 hypomethylated positions in GSE89702 (Figure 2(a)), 5087 hypermethylated positions and 4227 hypomethylated positions in GSE89703 (Figure 2(b)), 5116 hypermethylated positions and 4275 hypomethylated positions in GSE89705 (Figure 2(c)), and 5569 hypermethylated positions and 4801 hypomethylated positions in GSE89706 (Figure 2(d)). A total of 849 genes were identified as CDMGs shared by all four datasets (Figure 2(e) and [Supplementary-material supplementary-material-1]). However, these CDMGs were methylated at different sites and to different degrees in different brain regions.

### 3.2. CDMGs Involved in Multiple Neurological or Psychiatric-Related Biological Processes and Pathways

**Figure 3:**
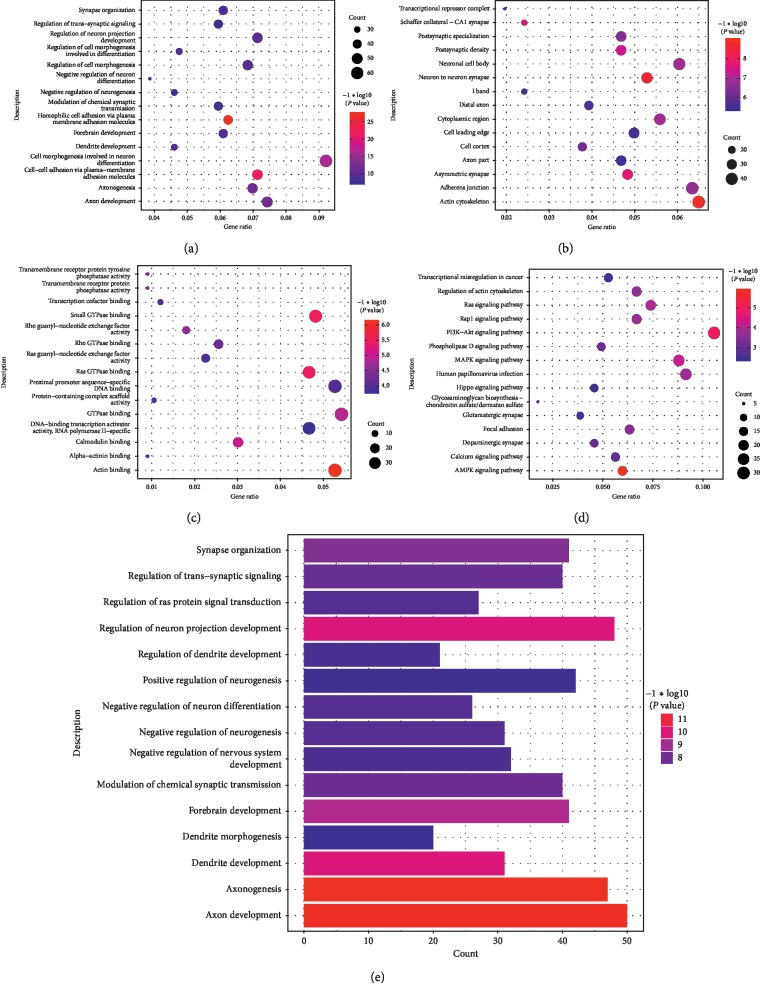
Functional and pathway enrichment analysis of genes. CDMGs were involved in gene ontologies including (a) cellular components, (b) biological processes, and (c) molecular functions. (d) KEGG pathway analysis for CDMGs. (e) The CDMGs most significantly involved in 15 neurological or psychiatric-related biological processes.

GO analysis of the SCZ-related CDMGs revealed these genes were involved in 244 biological processes, 43 cellular components, and 31 molecular functions (Figures 3(a)–3(d)). KEGG pathway analysis showed that the CDMGs were involved in 80 signaling pathways, most significantly in PI3K-Akt, AMPK, and m-activated protein kinases/extracellular regulated protein kinases (MAPK/ERK), as well as several pathways related to neurological or psychiatric-related biological processes (Figure 3(e)). Notably, the CDMGs were involved in multiple neuronal axon-related biological processes ([Supplementary-material supplementary-material-1]); this suggests that the methylation level of CDMGs in neuronal axons may be associated with SCZ.

### 3.3. DNA Methylation-Based Diagnostic Model for SCZ

**Figure 4:**
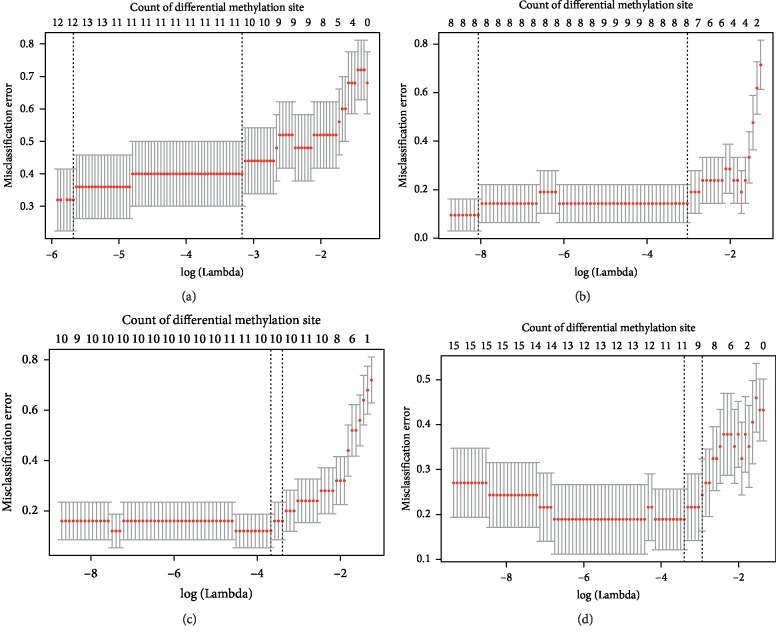
Cross-validation (10-fold) for tuning parameter selection in the LASSO model in the four datasets. (a) GSE89702. (b) GSE89703. (c) GSE89705. (d) GSE89706.

The following CDMGs were involved in the greatest numbers of neurological or psychiatric-related biological processes: SHANK3, WNT5A, NLGN1, GLI3, PTPRS, DISC1, SHH, BAIAP2, GLI2, and PAX6. The beta values of the differential methylation sites corresponding to these ten genes were used to construct a methylation template for diagnosing SCZ. Since our four datasets were taken from different regions of the brain, we found that these genes were differentially methylated based on their location—both in the brain region and on the DNA methylation position (Tables 1–4). Therefore, the beta values of the differential methylation sites corresponding to each gene were collected, and the LASSO method was used for variable selection and construction of the DNA methylation-based classifier. The results suggested that the counts of differential methylation sites selected by LASSO in different brain regions were various (Figure 4). The diagnostic ability of the same CDMG in different brain regions varied, which indicated that the epigenetic dysregulation of SCZ is complicated.

### 3.4. Diagnostic Efficiency of the Composite Model in Training and Validation

**Figure 5:**
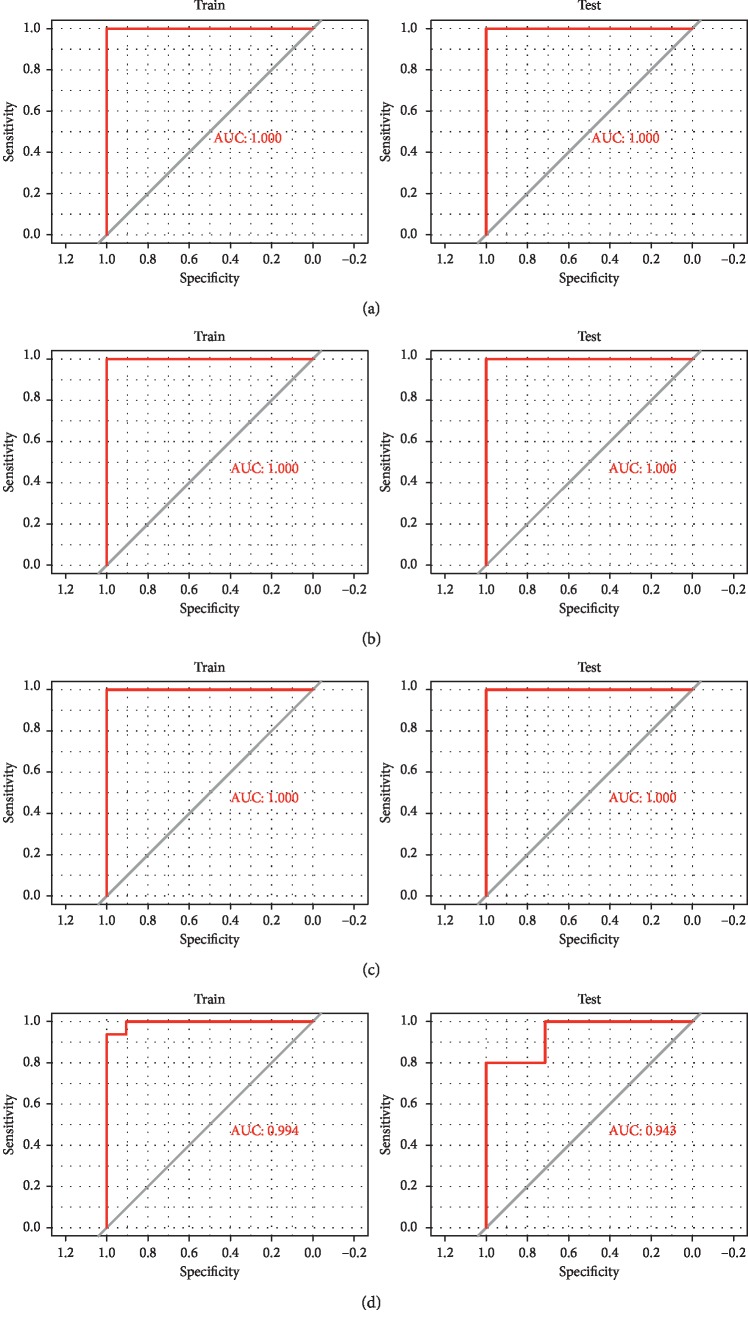
Receiver operating characteristic curve analysis of the ability of our DNA methylation-based classifier to identify schizophrenia in the training set (left) and the test set (right) in the four datasets: (a) GSE89702. (b) GSE89703. (c) GSE89705. (d) GSE89706.

In order to evaluate the diagnostic efficiency of the DNA methylation-based classifier, receiver operating characteristic curves were analyzed (Figure 5). In GES89702, GES89703, and GES89705, the accuracy, sensitivity, specificity, positive predictive value, negative predictive value, and AUC in the training and test sets were 1 (Tables 5–7). In GES89706, the accuracy was 0.950 in the training set and 0.920 in the test set, while the AUC was 0.994 in the training set and 0.943 in the test set (Table 8). The results suggest the DNA methylation-based classifier is a potential biomarker for diagnosing SCZ.

## 4. Discussion

In recent years, the morbidity and mortality rates of SCZ have increased, and the many health- and social-related problems for patients with SCZ are cause for much concern. However, the effective molecular diagnostic methods are unmet. In particular, diagnostic models that take into account both the molecular statistical and biological significance have not received much attention.

In the present study, a total of 849 CDMGs were identified in different brain regions. Functional enrichment analysis indicated the CDMGs were involved in various neurological or psychiatric-related biological processes and pathways, specifically signaling pathways PI3K-Akt, AMPK, and MAPK. The methylation levels of CDMGs may affect these biological processes and pathways. Our study identified biological processes with confirmed roles in mental diseases, including SCZ [20, 21]. MAPK/ERK and PI3K/Akt signaling pathways can regulate protuberant growth and protein synthesis related to neural plasticity, and it can assist in the normal development of nerve cells, which may protect against SCZ [22]. The initiation of nerve axon regeneration is regulated by the MAPK pathway and this initiates a neuronal response [23]. Further studies should explore directly whether the CDMGs in the present study are associated with SCZ.

The beta values of the differential methylation sites corresponding to 10 CDMGs (*SHANK*3, *WNT*5*A*, *NLGN*1, *GLI*3, *PTPRS*, *DISC*1, *SHH*, *BAIAP*2, *GLI*2, and *PAX*6) which were most often involved in neurological or psychiatric-related biological processes were used to construct a DNA methylation-based brain region-specific for SCZ. The methylation sites within genes and degree of methylation varied in different brain regions, suggesting that the methylation patterns of SCZ-related genes are extremely complex. The DISC1 protein regulates the development, maturation, and migration of brain neurons and synaptic signal transmission [24–27], and disruption can lead to various mental diseases, including SCZ [28, 29]. The process of nerve development and synaptic transmission regulated by DISC1 can be affected by its degree of methylation [30]. *SHANK*3 knock out may affect neuronal development and induce SCZ [31]. *SHANK*3 and *NLGN*1 are also related to the progression of SCZ [32, 33]. However, few reports exist on the association of genes *WNT*5*A*, *GLI*3, *PTPRS*, *SHH*, *BAIAP*2, *GLI*2, or *PAX*6 with SCZ. Our results suggest that the methylation level of these genes may be related to the disease. Indeed, our DNA methylation-based classifier showed strong diagnostic potential based on AUC analysis. It is worth noting that due to the characteristics of LASSO method, the more the inclusion variables, the better the effect of the classifier. However, from the perspective of economics, the more the variables (differential methylation sites) are included, the higher the cost is. So taking into account the effectiveness of the model and the cost of economics, we started to perform feature section and classifier construction from 10 genes instead of fewer or more. From the results of the present results, when we included 10 genes, LASSO method identified differential methylation sites of 7-8 genes for us and obtained the best AUC value (close to 1). So we may foresee that the inclusion of fewer genes may greatly reduce classification efficiency, while the inclusion of more genes may be not necessary because it would increase costs but not increase the effect.

Some critical limitations exist in the present study. Due to the small sample size, our DNA methylation-based SCZ diagnostic model needs to be further validated and improved in larger, independent datasets. The potential roles of CDMGs in SCZ need to be explored experimentally.

Despite these limitations, our findings suggest that gene methylation patterns are significantly associated with SCZ and may be a promising diagnostic method. Methylation levels and sites in CDMGs varied widely across different brain regions, and future studies should explore the potential relevance of this variation for SCZ onset and progression.

## Figures and Tables

**Table 1:** DNA methylation sites in GSE89702 for constructing the SCZ diagnostic model.

Probe ID	Genomic position	Gene	Gene region	*P* value	logFC
cg09615965	chr3:55522560–55522836	WNT5A	TSS1500	0.049	–0.012
cg09229620	chr3:122745183–122746986	NLGN1; NLGN1	5′UTR; 1st exon	0.048	0.038
cg16216907	chr7:107220344–107221075	GLI3	5′UTR	0.014	–0.052
cg10210571	chr1:231663999–231664608	TSNAX-DISC1	TSS200	0.039	–0.022
cg20841191		BAIAP2	Body	0.022	–0.031
cg26475541	chr17:79026043–79026355	BAIAP2	Body	0.022	–0.028
cg17454561	chr2:121745840–121746851	GLI2	Body	0.044	0.025
cg13570972	chr11:31839363–31839813	PAX6	TSS200	0.034	–0.015

FC, fold change; TSS1500, transcription start site 1500; TSS200, transcription start site 200; 5′UTR, 5′ untranslated region.

**Table 2:** DNA methylation sites in GSE89703 for constructing the SCZ diagnostic model.

Probe ID	Genomic position	Gene	Gene region	*P* value	logFC
cg09615965	chr3:55522560–55522836	WNT5A	TSS1500	0.049	–0.012
cg09229620	chr3:122745183–122746986	NLGN1; NLGN1	5′UTR; 1st exon	0.048	0.038
cg16216907	chr7:107220344–107221075	GLI3	5′UTR	0.014	–0.052
cg10210571	chr1:231663999–231664608	TSNAX-DISC1	TSS200	0.039	–0.022
cg20841191		BAIAP2	Body	0.022	–0.031
cg26475541	chr17:79026043–79026355	BAIAP2	Body	0.022	–0.028
cg17454561	chr2:121745840–121746851	GLI2	Body	0.044	0.025
cg13570972	chr11:31839363–31839813	PAX6	TSS200	0.034	–0.015

FC, fold change; TSS1500, transcription start site 1500; TSS200, transcription start site 200; 5′UTR, 5′ untranslated region.

**Table 3:** DNA methylation sites in GSE89705 for constructing the SCZ diagnostic model.

Probe ID	Genomic position	Gene	Gene region	*P* value	logFC
cg01777121	chr3:55517658–55517939	WNT5A	Body	0.044	0.028
cg21904441		NLGN1	Body	0.010	0.045
cg02709068		GLI3	Body	0.003	–0.029
cg06213317	chr19:5250458–5250668	PTPRS	Body	0.017	–0.038
cg27420590	chr19:5210564–5210864	PTPRS	3′UTR	0.023	–0.022
cg23476745	chr17:79077317–79077518	BAIAP2	Body	0.043	–0.028
cg23726427		GLI2	Body	0.032	0.022
cg26228577	chr2:121624827–121625209	GLI2	Body	0.010	–0.046
cg05490712		PAX6	5′UTR	0.031	–0.009
cg18058532		PAX6	5′UTR; 1st exon	0.041	0.007

FC, fold change; 3′UTR, 3′ untranslated region; 5′UTR, 5′ untranslated region.

**Table 4:** DNA methylation sites in GSE89706 for constructing the SCZ diagnostic model.

Probe ID	Genomic position	Gene	Gene region	*P* value	logFC
cg11676382	chr22:51142802–51143308	SHANK3	Body	0.031	–0.012
cg06304401		NLGN1	5′UTR	0.030	–0.007
cg19510409		NLGN1	Body	0.016	0.017
cg15078211	chr7:42267546–42267823	GLI3	5′UTR	0.011	–0.014
cg17390350	chr7:42267546–42267823	GLI3	5′UTR	0.002	–0.048
cg22411165		GLI3	Body	0.002	–0.020
cg10518481	chr19:5339640–5341061	PTPRS	TSS1500	0.002	0.026
cg04250837	chr1:231762414–231763115	TSNAX-DISC1	Body	0.041	0.019
cg07007152	chr7:155601175–155603235	SHH	Body	0.014	0.015
cg15244043	chr7:155604725–155605095	SHH	Body	0.007	0.011
cg20521696	chr7:155601175–155603235	SHH	Body	0.029	0.030

FC, fold change; TSS1500, transcription start site 1500; 5′UTR, 5′ untranslated region.

**Table 5:** Performance of DNA methylation-based classifier with GSE89702 data.

Cohort	Se	Sp	PPV	NPV	Accuracy	AUC
Training set	1.000	1.000	1.000	1.000	1.000	1.000
Test set	1.000	1.000	1.000	1.000	1.000	1.000

Se, sensitivity; Sp, specificity; PPV, positive predictive value; NPV, negative predictive value; AUC, area under the receiver operating characteristic curve.

**Table 6:** Performance of DNA methylation-based classifier with GSE89703 data.

Cohort	Se	Sp	PPV	NPV	Accuracy	AUC
Training set	1.000	1.000	1.000	1.000	1.000	1.000
Test set	1.000	1.000	1.000	1.000	1.000	1.000

Se, sensitivity; Sp, specificity; PPV, positive predictive value; NPV, negative predictive value; AUC, area under the receiver operating characteristic curve.

**Table 7:** Performance of DNA methylation-based classifier with GSE89705 data.

Cohort	Se	Sp	PPV	NPV	Accuracy	AUC
Training set	1.000	1.000	1.000	1.000	1.000	1.000
Test set	1.000	1.000	1.000	1.000	1.000	1.000

Se, sensitivity; Sp, specificity; PPV, positive predictive value; NPV, negative predictive value; AUC, area under the receiver operating characteristic curve.

**Table 8:** Performance of DNA methylation-based classifier with GSE89706 data.

Cohort	Se	Sp	PPV	NPV	Accuracy	AUC
Training set	0.890	0.900	1.000	0.900	0.950	0.994
Test set	1.000	1.000	0.800	1.000	0.920	0.943

Se, sensitivity; Sp, specificity; PPV, positive predictive value; NPV, negative predictive value; AUC, area under the receiver operating characteristic curve.

## Data Availability

The data used to support the findings of this study are included within Supplementary [Supplementary-material supplementary-material-1].

## Supplementary Materials

Supplementary Materials. Table S1: common differential methylated genes in four datasets. Table S2: common differential methylated genes involved in neurological or psychiatric-related biological processes.
