# Supplementary material for: A Novel Schizophrenia Diagnostic Model Based on Statistically Significant Changes in Gene Methylation in Specific Brain Regions
Source: Biomed Res Int. 2020 Feb 12;2020:8047146. doi: 10.1155/2020/8047146 (PMC7037884; doi:10.1155/2020/8047146)
Supplement: Supplementary Materials. — Table S1: common differential methylated genes in four datasets. Table S2: common differential methylated genes involved in neurological or psychiatric-related biological processes. [file 8047146.f1.zip › Table S1.docx]

| **Table S1. Common differential methylation genes in the four data sets** |
| --- |
| CDK15,WNT6,PTPRN2,GOLGA3,SLC20A2,TRIM15,CMIP,RERE,KIAA1549,RNF213,TNFAIP8L3,SDK1,KLHDC4,SLC12A7,SNTG2,IGF1R,SORCS2,PEX10,CLSTN1,ADAMTS17,MAFF,ANK1,WFDC1,DLGAP2,LRRC16A,RPS6KA2,FBXL18,MICB,DNAH17,PRDM16,CARD11,INS,IGF2,IQSEC3,CACNA1E,CUBN,EBF3,ODZ3,ATXN1,RNF220,NCOR2,FBRSL1,NPHP4,CALML4,IMMP2L,MACF1,3,VPS13B,PRKAG2,CHFR,RPTOR,C2orf3,VAT1L,TP73,CACNA1H,PPT2,NTRK1,INSRR,MGRN1,ZEB2,CYFIP1,LEPR,CAMKK2,PCDHGA4,PCDHGA2,PCDHGB2,PCDHGA1,PCDHGB1,PCDHGA3,SPTBN1,WDR27,NCALD,SETBP1,WHSC1,LRP1,TTYH3,FAM43A,CUX2,SPON1,LMF1,KCNT1,DLX1,MAD1L1,SND1,MAML3,ABR,IFT140,RIMBP2,DIP2C,NR5A2,TBC1D16,AHRR,TNXB,GJD3,WIPI2,MTUS2,TRIP12,SMAD9,PCDHA6,PCDHA2,PCDHA1,PCDHA9,PCDHA7,PCDHA5,PCDHA10,PCDHA3,PCDHA4,PCDHA8,VOPP1,PACS2,HEATR2,SDF4,F10,DSCAM,CDH13,GRHL3,KIAA0513,MACROD1,CDH3,NAV2,ZNRF3,DLG2,CCHCR1,TCF19,ZBTB22,TAPBP,AKAP13,GNAL,GRM4,ENOX1,MYO9B,ADARB2,KIAA1688,COL11A2,FOXP1,G3BP2,KIAA0182,MAGI2,ABTB2,LRP5,DAB2IP,C1orf94,CCDC36,FARP1,CELSR3,C7orf50,HS3ST4,TRIM27,PPP1R2P1,BANP,C6orf136,MIR548N,ARHGEF10,LPP,SHANK2,HOXC4,MCF2L2,RAP1GAP,IL17RA,RPH3AL,GFI1,TP53INP1,KCTD16,NOTCH4,FAT1,IGF2BP1,EIF2C2,SLC6A18,ARL15,CTNND2,CPLX2,LOC285768,PCGF3,GAK,ALG5,AGPAT4,PRKAR1B,CYTH3,ALOX5,RGL1,RTN4RL1,ZNF193,GPR133,MPRIP,AATK,SMOC2,ACCN4,MYT1L,INPP5A,ERICH1,SOX2OT,CD81,KANK3,FBXW8,CTXN1,HCCA2,GABRG3,MIR548F5,DCLK1,LMO3,ACOT7,COL23A1,LMX1A,MED16,APBB2,TBCD,TFAP2A,RORA,TTC15,CHPF,PLEKHA6,CALD1,SIM2,ASTN2,PCDHGB5,PCDHGA6,PCDHGA8,PCDHGA5,PCDHGB4,PCDHGA7,PCDHGB3,LOC440356,TRIM31,ZNF529,SHH,JAKMIP3,OTX1,RADIL,GLB1L2,ATP11A,C3orf21,ZFPM1,MAGI1,KLHL29,RBMS1,HPCAL1,GLI3,ATP13A1,CUX1,CUGBP2,ZBTB4,SEPT9,SYNGAP1,OBSL1,TSSC1,PRDM8,CLMN,MICAL3,AZIN1,NEXN,TSNAX,DISC1,CDH23,EGFR,SH3RF3,BAIAP2,LHFP,VAV2,TPO,MGAT5B,THRB,BRD2,AGPAT1,ANKRD11,LOC399959,HOXC5,HOXC6,DAXX,RASA3,KIRREL3,KIAA1026,KCNQ1,FKBPL,HDAC4,VPS52,GAS7,PTPRS,C2CD4C,SOX5,SPEN,PPP2R2B,FBXO31,FLJ22536,B3GNTL1,LRBA,AGBL4,DST,FEN1,PTPRG,TEKT3,CYB5R2,TMC6,MOV10L1,PTPRU,GRIN2A,PLEC1,SDCCAG8,TBX15,WDR8,SRI,CSGALNACT1,CXXC5,TRAPPC9,MAP7D1,SORBS2,ODZ4,CTBP1,IGSF21,EHMT2,LOC645323,ITPK1,GPC6,WSCD1,DPP10,STK32C,PRDM9,GRIK3,SGCE,PEG10,BAG5,RGS12,TMEM132D,SLC22A23,RGL2,SCAND3,KIAA1324,WDR46,SNX29,ILDR2,BAT2,PALLD,FLJ43860,GALNTL4,MBNL2,TMTC1,OBSCN,PIK3C2B,RAD51L1,PARD3,IL17RD,UNKL,PUM1,KCNN2,AGAP1,TCERG1L,PRDM15,PTPRA,NTM,MAPK8IP3,FOXK1,SIN3A,LAMA2,TCF12,CACNA2D4,TSNARE1,COL6A3,PSORS1C1,RBMS3,SNORD115,C1orf92,NRG1,TSC2,COL5A1,LRPAP1,BCL9,LOC100130987,LOC404266,PDGFD,CDH8,GDF6,LAMA4,KIF17,HSPD1,HSPE1,ADAMTS2,PTPRF,OPCML,MEG3,HIVEP3,ABLIM2,CRMP1,TGIF1,SIX3,NFATC1,PRPH,RPS18,HLA,IGFBP5,FLOT1,PTER,ARHGEF4,C3orf26,FILIP1L,MIR548G,GMDS,SSU72,EHBP1,GPSM3,EPHA7,DLC1,C6orf25,ABCF1,MAFK,TLL1,KCTD1,SLC38A10,BCL2L11,PPP2R2D,BDNF,DNM3,GREM2,SYT5,2,COX6B2,FAM71E2,FAM172A,SDK2,RAB7L1,PPP2R3A,PDGFB,B3GNT7,KIAA1949,IPO11,MYO10,PBX1,ADD2,SGMS1,C1orf114,CAMTA1,MYO1B,TRIO,SRGAP3,SLC9A3,PAX3,CCDC140,RGS7,SH2D4B,SLC6A19,TLR5,TLE3,INPP4A,COL13A1,CDC7,ST6GALNAC3,TRRAP,PAX7,MKLN1,PHACTR1,HTRA1,FAM38A,IQCE,ARHGEF7,BCL11B,RAPGEF5,STARD13,NXPH1,GPC5,CCDC149,FILIP1,RAB1B,MYLK,FLT4,VWA3B,LRRC61,AKAP12,KLF13,MAFG,MS4A13,GNL1,DSCAML1,PPP3CB,KLF14,CHST8,C1orf159,ZBBX,NUAK1,MAP2K5,ADPRHL1,SLC15A4,AP2A2,BRF1,ALOX12P2,MYO1E,BCL11A,ARMC5,PHF1,PTK2B,SLC44A4,NXN,ZC3H13,LONP1,PVT1,TMEM200B,NRM,SYT7,MEST,ZBED3,WNT5A,SKI,LMNA,RBP1,ITPR1,CDC42BPB,APBA2,CSMD1,OLFM3,DHX29,SKIV2L2,TTLL5,CRYL1,ITPKA,CLPP,MORN1,PRDM2,DRD4,PFKP,AFAP1,ECE2,C11orf41,TBC1D22A,CYP4V2,RELT,PACRG,LOC285796,SYT13,CPXM2,LOC100133612,SHANK3,MMS19,UBTD1,SBNO2,DAB1,21,SLIT1,AMN1,DGCR14,GATA4,GIGYF2,ISPD,LHX8,GNAS,FERMT2,GNG12,MTDH,CHST11,DCTN6,FGFR2,CACNA1I,RAI1,ZKSCAN4,KIAA0922,FRMD4A,GLI2,IFFO2,NECAP2,HOXC9,CEP72,NDUFA4L2,DNAH2,DDAH2,MESTIT1,TMEM116,ERP29,EXOC4,CREBBP,PTDSS2,METTL3,RARG,ATP2A1,FLJ41350,RBM28,STOX2,AFF3,COL6A6,C11orf87,PTRF,TSHZ1,PARVB,LTBP2,CR1L,NCLN,SMTNL2,VTRNA1,SSH1,MAN2B2,GPR98,EXOC3,NINJ2,RNF212,ACACA,CACHD1,PRKCZ,MEGF11,BTNL2,PKM2,SORCS3,DIAPH3,PARVG,RAB40B,MIR668,MIR485,LMTK2,SLC2A5,HOPX,PLXNB1,ACBD7,TEAD1,NEURL,MCF2L,SRCIN1,ZAK,PNPLA1,P4HTM,FAM73B,AJAP1,ELFN1,PPP2R5C,RUNX1,SH3BP4,JMJD6,PHLDB3,TRIM29,TRPV3,LHX6,ZNF384,FOXO3,ZBTB20,RBM20,REXO1,TERT,NRXN1,KNDC1,GLT1D1,PPAP2C,TLN2,SDCBP2,OR8U8,SERPINE2,ABCB1,GABBR1,GALNT9,MIR453,FAM19A5,FAM171A1,KCNS3,SNORD116,RASIP1,1,OTUD7B,AUTS2,KLF5,LHX5,DIP2B,FOXK2,CYP26B1,ARID3A,GNASAS,NOTCH3,TET1,CTTN,PITPNA,DGKI,GJB3,NLGN1,UBR4,PTPRE,CASZ1,ZCCHC3,RECQL5,ITGAE,BAT4,CIT,KRT7,SRRM4,TRIM62,C1orf183,MGMT,ARL5C,HPSE2,PCDH21,MYH11,LOC100294362,RAP2A,DNAH7,TFEB,CDH22,GPR4,SBK1,DOA,ERI3,TRIM26,MIR548H4,CD84,BRSK2,FYN,RALGAPA2,KLHL30,FRAS1,ATM,NPAT,ZNF827,GPR108,ATXN7L1,LHX3,SLC6A13,CHD5,CCNA1,MPPED2,CSGALNACT2,UBTF,FAM188B,CPLX1,FAM101A,DYSF,LRRK1,NEU4,KLF3,KDM2A,VCL,PPARG,CDC42BPG,C7orf20,PCNT,MAP2K4,LDB2,GNG8,UST,TCF21,ASCL2,C11orf63,8,DPF3,C1orf53,MRGPRF,UBAC2,SMYD3,L3MBTL,MYOM2,MPPED1,ALK,FAT3,MYCN,CD96,TBRG4,PCIF1,HIC1,PAX6,P2RX3,9,MLH1,EPM2AIP1,PCDHGA9,PCDHGB6,PCDHGB7,PCDHGA10,NGF,MAN2A1,OCA2,DKK3,ADAMTS10,CPNE5,AFF1,MUC5B,FLNB,CDYL,TUBA8,SHPRH,PTDSS1,TBC1D7,KIRREL2,PEX14,MAD2L2,NAT15,DCTN4,VGLL4,SSBP3,LUZP6,MTPN,GNMT,STAMBPL1,GFPT2,NEDD9,BBX,GTDC1,SF3A1,TBL2,LOC285375,SNHG3,RCC1,KRT79,GDF7,CRTC2,RNF39,DNAJB6,INSC,DLGAP1,ALX4,DLEU2,HS3ST3A1,ST6GAL1,MEAF6,PRMT1,AZI1,MTA1,SLC6A16,FYCO1,BMPR1A,CHRM2,PYGM,C2orf85,TECTA,BAT1,RHOU,LOC550112,UBA6,CEP350,LOC285830,HYAL3,NAT6,AGBL1,CACNB2,SYT3,MIRLET7B,LOC400931,SRR,PSORS1C3,COLEC11,PCDHA12,PCDHA13,PCDHA11,ARPC1B,TLE2,CRTC1,ERC1,WDR1,MYO15A,STK19,DOM3Z,RAVER2,C6orf47,NOX4,LOC646982,C2CD4A,TRAK1,GPR123,TOMM40L,RARA,EXOC2,TTN |
